# Effects of compound probiotics on the weight, immunity performance and fecal microbiota of forest musk deer

**DOI:** 10.1038/s41598-019-55731-5

**Published:** 2019-12-16

**Authors:** Xu Liu, Wei Zhao, Dong Yu, Jian-Guo Cheng, Yan Luo, Yin Wang, Ze-Xiao Yang, Xue-Ping Yao, Shao-Shuai Wu, Wu-You Wang, Wei Yang, Dan-Qin Li, Yi-Ming Wu

**Affiliations:** 1 College of Veterinary Medicine, Sichuan Agricultural University, Wenjiang, Sichuan China; 2 Sichuan Institute of Musk Deer Breeding, Dujiangyan, Sichuan China

**Keywords:** Innate immunity, Applied microbiology

## Abstract

Probiotics are intended to provide health benefits when consumed, generally by improving or restoring the gut flora. The health problems of forest musk deer (FMD, *Moschus berezovskii*), a threatened species currently under conservation, restrict the development of captive musk deer. This study was conducted with the aim of analyzing the effects of forest musk deer compound probiotics (FMDPs) on weight, immunity performance and fecal microbiota in FMD by measuring average daily weight gain (ADG) and immune-related factors and by using high-throughput 16S rRNA sequencing to investigate differences in the fecal microbiota among the control group (4 samples), treatment group A (4 samples) and treatment group B (4 samples). The results showed that the ADG of treatment groups A and B was significantly higher than that of the control group (*p* = 0.032, *p* = 0.018). The increase in IgA and IgG levels in treatment group B was significantly higher than that in the control group (*p* = 0.02, *p* = 0.011). At the phylum and genus levels, the difference in bacterial community structure was significant between treatment group B and the control group. Both the alpha diversity and beta diversity results showed significant differences in the microbiota of FMD before and after FMDP feeding. In summary, the results indicated that FMDPs could promote the growth of growing FMD, improve immunity and balance the role of intestinal microbes.

## Introduction

Probiotics are live microorganisms that benefit the hosts by gut-colonizing. Controlling the composition of the microflora through the intake of probiotics is an attractive method that works by regulating the fecal microbiota to help maintain and restore health^1^. Probiotics can alter the intestinal flora by reducing the pH of the cavity, competing for nutrients, secreting antibacterial compounds (organic acids, biosurfactants, hydrogen peroxide, bacteriocins, etc.) to prevent pathogenic bacteria from adhering and invading epithelial cells and inducing the production of antibacterial compounds^2–4^.

In particular, *Lactobacillus* species have strong bactericidal effects on some pathogenic bacteria. One of the most important underlying mechanisms known is the inhibition of the activity of pathogenic bacteria by the metabolites of *Lactobacillus* species. Some inhibitors have been identified, including organic acids^5^. This type of acidic substance can reduce the surrounding pH and effectively inhibit the growth and reproduction of various pathogenic bacteria but has no influence on the surrounding probiotics and can eventually establish a microecological environment dominated by probiotics in the intestinal tract^6^. This mechanism has been confirmed by Marwati and Cahyaningrum who performed the bacteriocin activity test on the natural supernatant of *Lactobacillus* SCG 1223 and found the bacteriocin produced by *Lactobacillus* SCG 1223 could inhibit the activity of *L. monocytogenes*, *S. thypimurium* and *E. coli*.^7^

Probiotics have broad applications in ruminant animals, such as cows, sika deer and lambs^8^. As probiotic feed additives, *Bacillus* species, such as *Bacillus licheniformis*, and *Lactobacillus plantarum* have been widely used in cattle raising; these species can improve feed quality and feed utilization rate, promote animal growth and even prevent disease^9,10^. Keles *et al*.^11^ investigated the effects of various combinations of lactic acid bacteria on the conservation characteristics of lamb performance and confirmed that *Lactobacillus buchneri* could provide suitable conditions for inhibiting the activity of harmful microorganisms.

Wild Forest musk deer (FMD) are endangered because of poaching for musk, which is an extremely valuable substance produced by male FMD. FMD is included as the Endangered Species (EN) in The IUCN Red List and in Appendix II of the Convention on International Trade in Endangered Species of Wild Fauna and Flora (CITES). FMD is also included in the National Register of Key Protected Wild Animals: National Level Protected Animals^12–15^. For sustainable use of musk resources, musk deer breeding farms have been developed since 1958 in China. After years of unremitting efforts, some progress has been achieved in captive forest musk deer^16,17^, and wild FMD populations are also recovering with legal protection. In our previous report, FMD compound probiotics (FMDPs) were developed and tested in mice^18^. However, FMDP has never been studied in FMD directly.

To better understand the probiotic function of compound probiotics in FMD, fresh feces were collected from the control group and treatment groups in the same period. Immune-related factors were monitored by an enzyme-linked immunosorbent assay (ELISA) and the diversity of the FMD intestinal microbiota was investigated by high-throughput 16S rRNA sequencing technology.

## Results

### Determination of body weight

**Figure 1:**
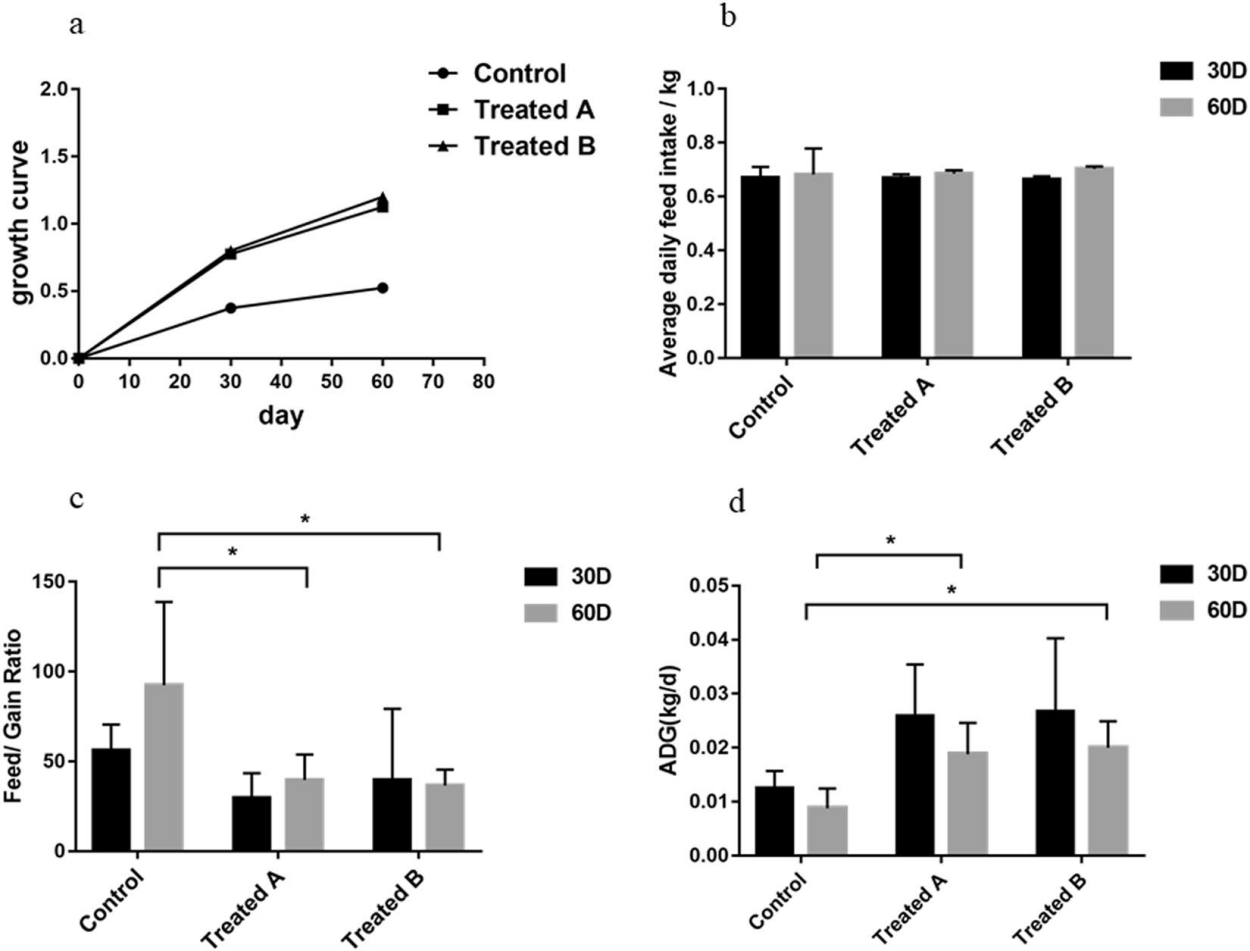
Differences in FMD weight (mean ± SD) between the control and treatment groups. The significance of the differences among the three groups was determined using the independent-sample t-test *P < 0.05.

The differences in weight between FMD fed basal diets and FMD fed FMDPs are shown in Fig. 1. On day 30 and day 60, there were no significant differences in the average daily feed intake of the control group and treatment groups. On day 30, there was no significant difference in the feed/gain (F/G) ratio between the control group and treatment groups. On day 60, the F/G ratio of the treatment groups was significantly higher than that of the control group (*p* < 0.05). As shown in the growth curve, from day 0 to day 30, the three groups grew rapidly, and the growth rate of the three groups decreased from day 30 to day 60. The growth rate of the treatment groups was higher than that of the control group, and the average daily weight gain (ADG) of the treatment groups was significantly higher than that of the control group on day 60 (*p* < 0.05).

### Determination of immune-related factors

**Figure 2:**
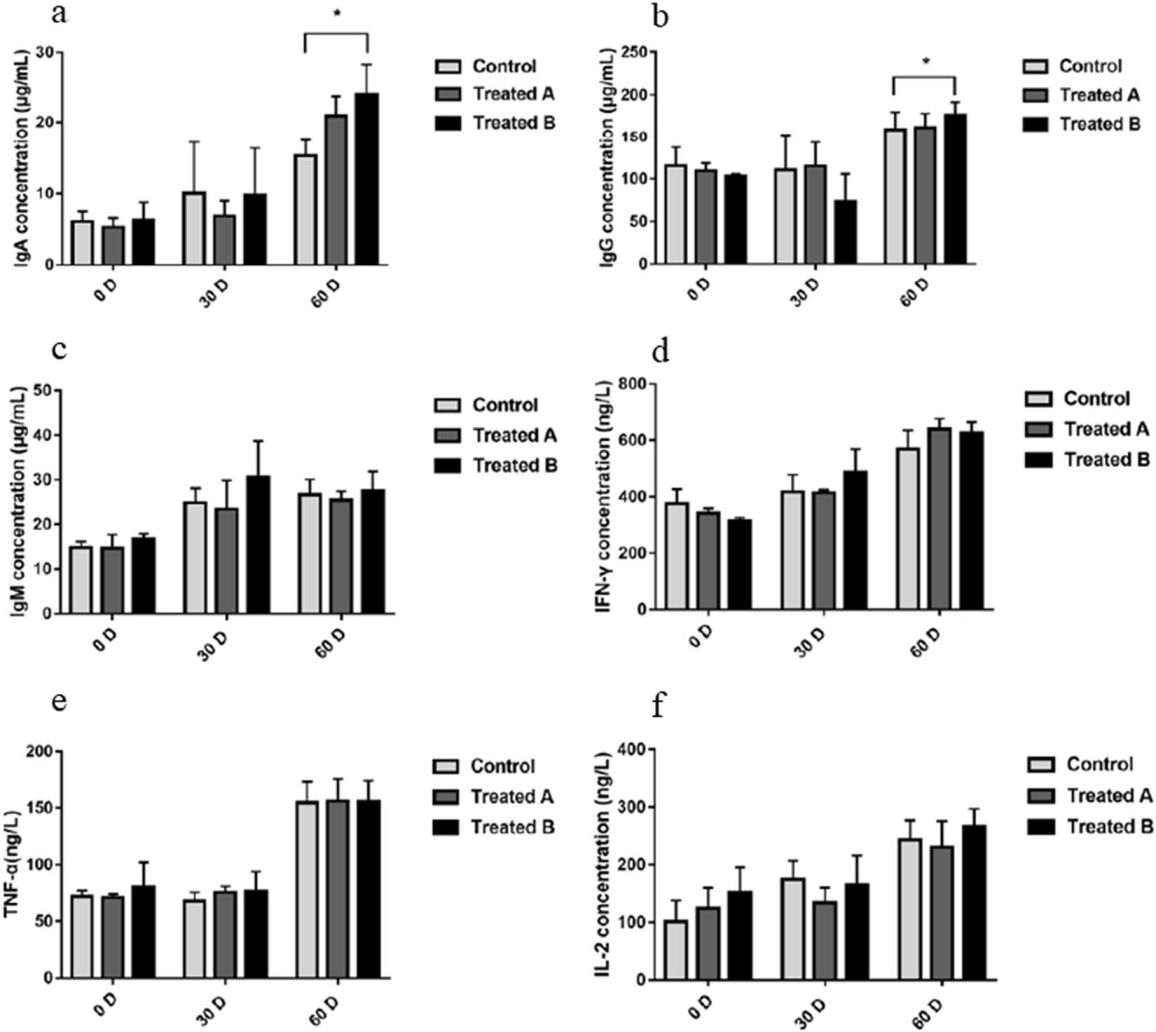
Differences in immunoglobulin indices of FMD between the control and treatment groups. The significance of the differences among the three groups was determined using the independent-sample t-test *P < 0.05.

The differences in immune-related factors between FMD fed basal diets and FMD fed FMDPs are presented in Fig. 2. The increase in IgA and IgG levels in treatment group B was significantly higher than that in the control group (*p* < 0.05), and there was no significant change in TNF-α, IFN-γ, IgM and IL-2 levels (*p* > 0.05).

### Determination of the number of lactic acid bacteria in feces

**Figure 3:**
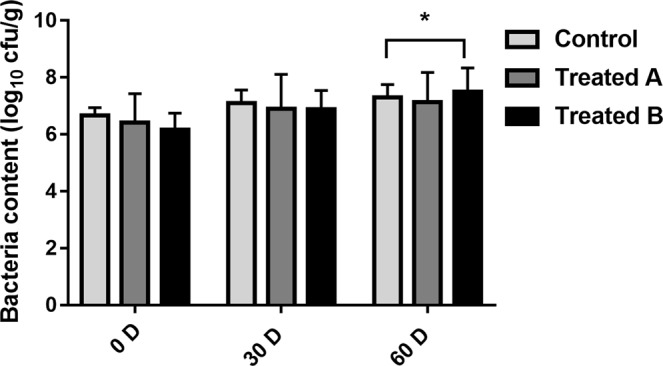
Differences in the number (mean ± SD) of lactic acid bacteria in feces of FMD between the control and treatment groups. The significance of the differences among the three groups was determined using the independent-sample t-test *P < 0.05.

The number of lactic acid bacteria increased in the feces of all three groups. While the number of lactic acid bacteria in treatment group B was significantly higher than that in the control group (*p* < 0.05), there was no significant difference between treatment group A and the control group (Fig. 3).

### Analysis of 16S rRNA sequencing results

The taxonomic information for each operational taxonomic unit (OTU) was obtained by comparing the representative OTU sequence with the template sequence from the Greengenes database^19^.

The bacteria that could be tested were classified into 21 phyla, 37 classes, 63 orders, 132 families, and 256 genera. In Table 1, “Phylum”, “Class”, “Order”, “Family” and “Genus” correspond to the number of OTUs that could be classified at that level in each sample.

**Table 1:** Top 3 phyla in terms of relative abundance among the three groups.

Sample	Top 1	Top 2	Top 3
A1	*Firmicutes* (49.49%)	*Proteobacteria* (30.04%)	*Bacteroidetes* (9.90%)
B1	*Firmicutes* (39.19%)	*Proteobacteria* (43.08%)	*Bacteroidetes* (8.63%)
C1	*Firmicutes* (41.43)	*Proteobacteria* (41.63%)	*Bacteroidetes* (7.82%)
A2	*Firmicutes* (50.78%)	*Proteobacteria* (30.04%)	*Bacteroidetes* (9.90%)
B2	*Proteobacteria* (45.37%)	*Firmicutes* (38.02%)	*Bacteroidetes* (8.35%)
C2	*Proteobacteria* (54.75%)	*Firmicutes* (29.53%)	*Bacteroidetes* (10.25%)
A3	*Firmicutes* (66.27%)	*Bacteroidetes* (18.25%)	*Verrucomicrobia* (4.49%)
B3	*Firmicutes* (61.20%)	*Bacteroidetes* (18.09%)	*Verrucomicrobia* (5.68%)
C3	*Firmicutes* (66.36%)	*Bacteroidetes* (17.83%)	*Actinobacteria* (4.42%)

A1–A3 represent the control group on day 0, day 30, and day 60; B1–B3 represent treatment group A on day 0, day 30, and day 60; C1–C3 represent treatment group B on day 0, day 30, and day 60.

### Comparison of the fecal microbiota of FMD before and after FMDP feeding

**Figure 4:**
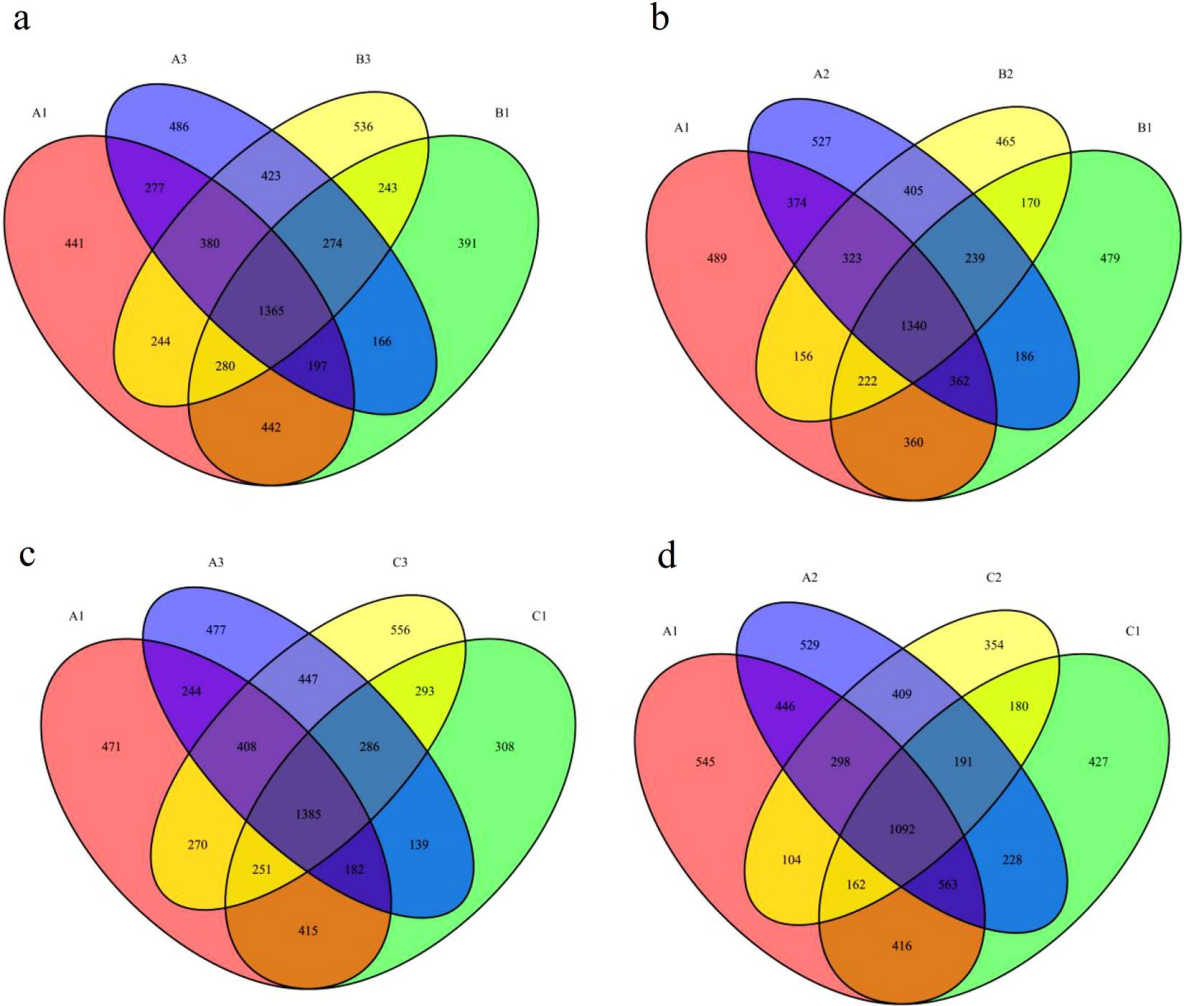
Venn diagram. The Venn diagrams show the number of OTUs that were shared or not shared by the control group and treatment group individuals, depending on overlap. For this presentation, two individuals had to be combined, thereby reflecting the number of OTUs shared by both individuals. (**a**) The number of OTUs shared by A1, B1, A2 and B2. (**b**) The number of OTUs shared by A1, C1, A2 and C2. (**c**) The number of OTUs shared by A1, B1, A3 and B3. (**d**) The number of OTUs shared by A1, C1, A3 and C3. A1–A3 represent the control group on day 0, day 30, and day 60; B1–B3 represent treatment group A on day 0, day 30, and day 60; C1–C3 represent treatment group B on day 0, day 30, and day 60.

A Venn diagram was used to determine the core fecal microbiota among the three groups and was presented in Fig. 4. The components shared by all individuals in each group were considered to be the core bacterial communities. On day 0, there were 2284 OTUs shared by group A1 and group B1, the number of unique OTUs in group A1 was 1342, and the number of unique OTUs in group B1 was 1074. There were 2233 OTUs shared by group A1 and group C1, the number of unique OTUs in group A1 was 1393, and the number of unique OTUs in group C1 was 1026. On day 60, there were 2442 OTUs shared by group A3 and group B3, the number of unique OTUs in group A3 was 1126, and the number of unique OTUs in group B3 was 1303. There were 2526 OTUs shared by group A3 and group C3, the number of unique OTUs in group A3 was 1042, and the number of unique OTUs in group C3 was 1370. On day 60, the number of components shared by the control group and treatment group A increased, and the number of components shared by the control group and treatment group B was higher than that on day 0. While the number of unique OTUs in the control group on day 60 was lower than that on day 0, the number of unique OTUs in the treatment groups was higher than that on day 0.

### Diversity analysis of the FMD microbiota before and after FMDP feeding

**Figure 5:**
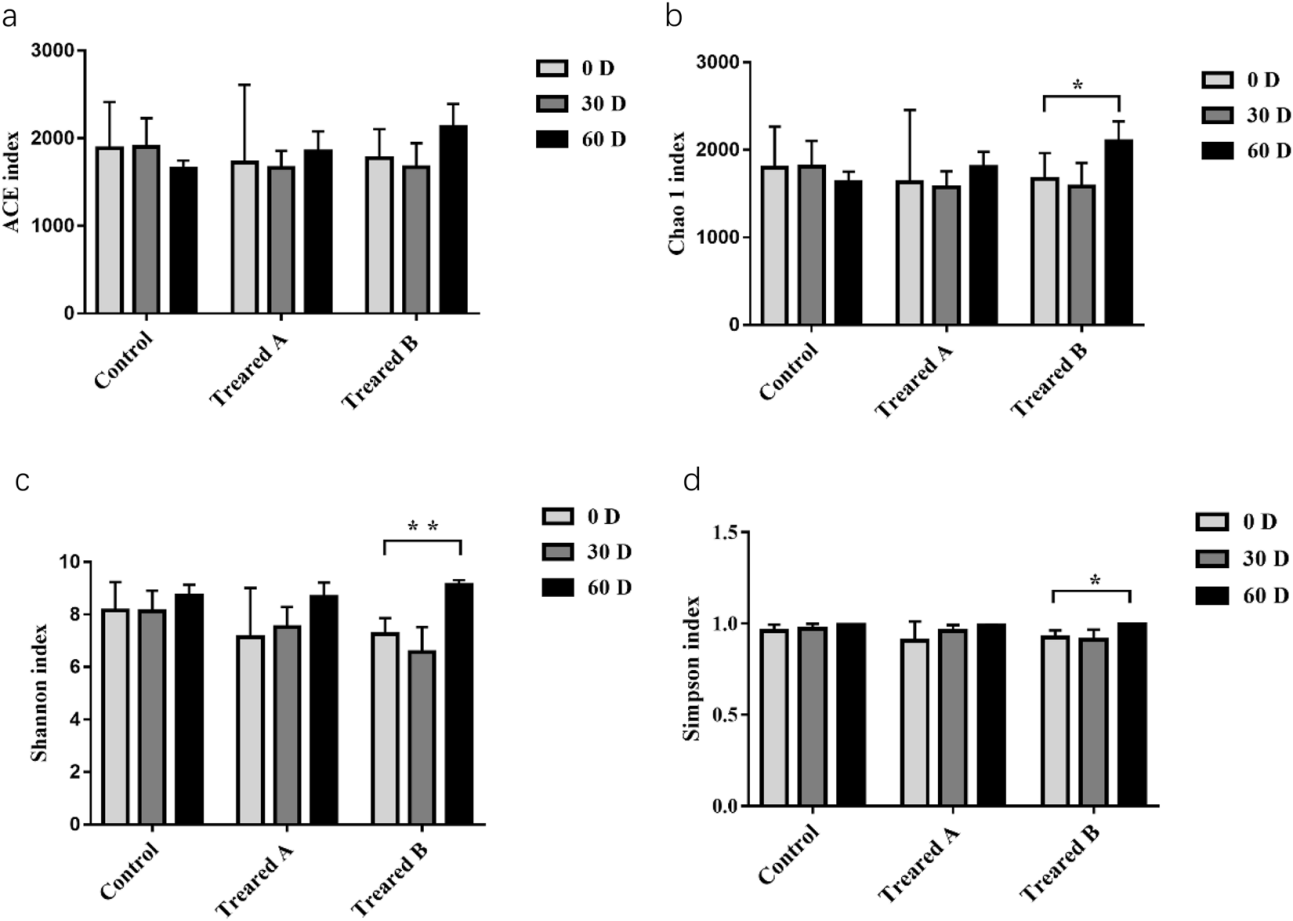
Comparison of alpha diversity indices of the fecal microbiota in the control and treatment groups. (**a**) ACE: an index used to estimate the number of OTUs in a community. (**b**) Chao: an index that uses the Chao1 algorithm to estimate the number of OTUs included in a sample. Chao is commonly used in ecology to assess the total number of species. (**c**) Shannon: an index often used to reflect alpha diversity and estimate microbial diversity in a sample. (**d**) Simpson: a diversity index commonly used in ecology to quantitatively describe the biodiversity of a geographical area. *P < 0.05. **P < 0.01.

Alpha diversity indices (including the ACE, Chao1, Shannon, and Simpson indices) reflect the richness and diversity of a single sample species. The Chao1 and ACE indices measure species richness, while the Shannon and Simpson indices represent species diversity. As shown in Fig. 5, the Chao1 index of treatment group B increased after FMDP feeding, and the difference was statistically significant (*p* < 0.05), but there was no significant change in the ACE index. There was no significant change in the Chao1 and ACE indices of the control group and treatment group A after FMDP feeding (*p* > 0.05), but the Chao1 and ACE indices of the control group both showed a downward trend. The Shannon and Simpson indices of treatment group B both increased after FMDP feeding, and the difference was statistically significant (*p* < 0.05); the Shannon index showed a marked difference (*p* < 0.01). There were no significant changes in the Shannon and Simpson indices of the control group and treatment group A (*p* > 0.05).

**Figure 6:**
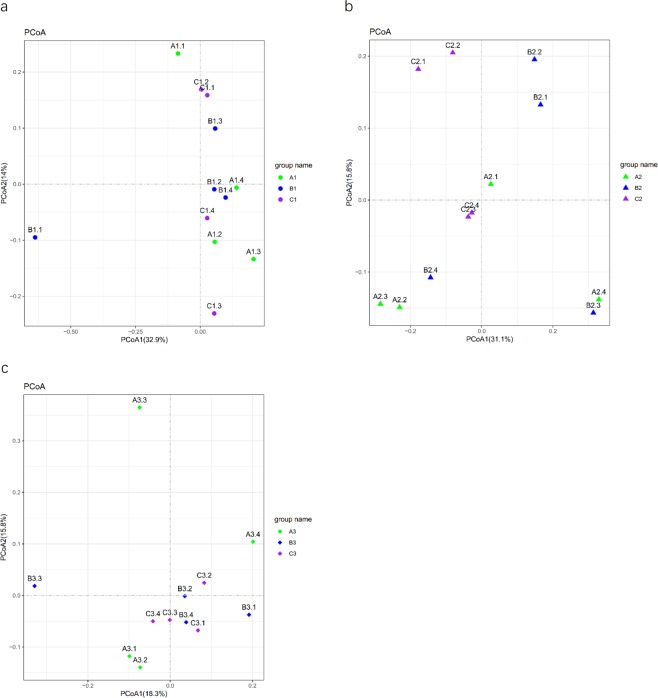
Principal coordinate analysis (PCoA) plot. Different colors indicate different groups, the color Green represents control group samples, the color blue represents treatment group A samples, and the color purple represents treatment group B samples. Samples in the same group are represented by the same color and shape. The distances between the sample points represent the similarity of the microbiota in the samples. The shorter the distance is, the higher the similarity, and samples that cluster together are composed of similar microbes. (**a**) The bacterial community structure of FMD on day 0. (**b**) The bacterial community structure of FMD on day 30. (**c**) The bacterial community structure of FMD on day 60.

Principal coordinate analysis (PCoA) was used to show the natural distribution of the sample at a certain distance scale^20^, reducing the data structure by decomposing the sample distance matrix. Each point represents a sample, and different colors represent different samples or groups. The distance between two points is in direct proportion to the difference of the microbial community structure between the two samples and in inversely proportional to the similarity. As observed in the PCoA results shown in Fig. 6, samples after two FMDP feedings exhibited obvious clustering.

### Analysis of the differences in the FMD fecal microbiota before and after FMDP feeding at the phylum and genus levels

**Figure 7:**
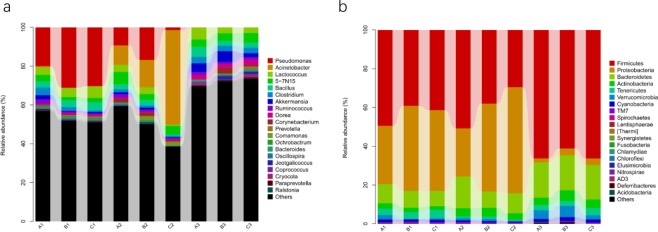
Fecal microbial composition of the control and treatment groups at the phylum and genus levels. (**a**) Phylum level. (**b**) Genus level. Each bar represents the top twenty bacterial species ranked by the relative abundance in each individual sample or group.

The differences in relative abundances of the top 20 bacterial communities at the phylum and genus levels in the control and treatment groups are shown in Fig. 7.

The top 3 phyla in terms of relative abundance among the three groups are shown in Table 1. On day 0, *Firmicutes* was the predominant phylum in all three groups, followed by *Proteobacteria* and *Bacteroidetes*. On day 30, the top 3 phyla in terms of relative abundance in the control group were *Firmicutes*, *Proteobacteria* and *Bacteroidetes*, while those in the treatment groups were *Proteobacteria*, *Firmicutes* and *Bacteroidetes*. On day 60, the top 3 phyla in terms of relative abundance in the control group and treatment group A were *Firmicutes*, *Bacteroidetes* and *Verrucomicrobia*, while those in treatment group B were *Firmicutes*, *Bacteroidetes* and *Actinobacteria*.

At the phylum level, on day 30, the relative abundance of *Tenericutes* was lower than that on day 0 in the control group and treatment group B. On the other hand, on day 30, in treatment group B, the relative abundance of *Fusobacteria* was lower than that on day 0, and in treatment group A, the relative abundance of *Spirochaetes* was lower than that on day 0. The relative abundance of *Proteobacteria* was significantly lower on day 60 than on day 0 in all three groups (*p* < 0.05), while in treatment group B, the relative abundances of *Firmicutes* and *Verrucomicrobi*a on day 60 were significantly higher than those on day 0 (*p* < 0.05). Compared with the control group, the F/B ratio increased in treatment group B with the increase in *Firmicutes* abundance (*p* < 0.05).

At the genus level, the relative abundance of *Adlercreutzia* was higher on day 30 than on day 0 in the control group (*p* < 0.05). On day 30, the relative abundances of *Leuconostoc* and *Coprococcus* were lower than those on day 0 in treatment group A (*p* < 0.05), while in treatment group B, the relative abundance of *Acinetobacter* was higher than that on day 0 and the relative abundances of *Pseudomonas*, *Coprococcus* and *Ruminococcus* were lower than those on day 0 (*p* < 0.05). On day 60, the relative abundance of *Pseudomonas* in all three groups was significantly decreased (*p* < 0.05), the relative abundance of *Enterococcus* in treatment group A was significantly decreased (*p* < 0.05) and the relative abundances of *Ruminococcus*, *Akkermansia*, *Oscillospira*, *Coprococcus* and *Clostridium* in treatment group B were significantly increased (*p* < 0.05). (Table 2)

**Table 2:** Significantly different phyla and genera among the three groups.

	Group VS Group	Phylum & Genus	Δmean	P value
Phylum	A1 VS A2	*Tenericutes*	−0.023217	0.00775
	B1 VS B2	*Spirochaetes*	−0.00065	0.0278
	C1 VS C2	*Tenericutes*	−0.038678	0.002429
		*Fusobacteria*	−0.000136	0.021214
	A1 VS A3	*Proteobacteria*	−0.280022	0.019429
	B1 VS B3	*Proteobacteria*	−0.399961	0.009437
		*Lentisphaerae*	0.000191	0.007687
	C1 VS C3	*Proteobacteria*	−0.383878	0
		*Firmicutes*	0.249605	0.011933
		*Verrucomicrobia*	0.012974	0.0246
Genus	A1 VS A2	*Adlercreutzia*	0.010953	0.035256
	B1 VS B2	*Leuconostoc*	−0.000764	0.016433
		*Coprococcus*	−0.006022	0.046817
	C1 VS C2	*Acinetobacter*	0.719646	0
		*Pseudomonas*	−0.565002	0.00068
		*Coprococcus*	−0.003435	0.018864
		*Ruminococcus*	−0.002862	0.027417
	A1 VS A3	*Pseudomonas*	−0.405009	0.005293
	B1 VS B3	*Pseudomonas*	−0.577228	0.000882
		*Enterococcus*	−0.000336	0.006953
	C1 VS C3	*Pseudomonas*	−0.58603	0.000744
		*Ruminococcus*	0.026891	0.002561
		*Akkermansia*	0.051469	0.003902
		*Oscillospira*	0.009757	0.003378
		*Coprococcus*	0.009746	0.030085
		*Clostridium*	0.076048	0.026622

A1–A3 represent the control group on day 0, day 30, and day 60; B1–B3 represent treatment group A on day 0, day 30, and day 60; C1–C3 represent treatment group B on day 0, day 30, and day 60.

Δmean represents the difference in the mean between the two groups.

## Discussion

Many studies have shown that probiotics can increase weight^21,22^. A study on the effects of complex probiotics (*B. licheniformis* and *Bacillus subtilis*) on the weight of calves showed that addition of probiotics to the diet increased the ADG and body mass index of calves aged 0–8 weeks^23^. Jia *et al*.^24^ found that using *B. licheniformis* and *Saccharomyces cerevisiae* in fattening lambs could improve the ADG. In this study, the growth rate of the treatment groups was higher than that of the control group, the F/G ratio of the treatment groups was significantly decreased, and the ADG of treatment groups was significantly increased on day 60. However, there were no significant differences in the average daily feed intake between the control group and treatment groups. The results indicated that FMDPs might promote the growth of FMD.

It is well known that the fecal microbiota maintains the host’s digestive and immune systems. Several studies have declared that probiotics have beneficial effects on immune function in animals^25–28^. The intestinal epithelial cells (IECs) and dendrite cells (DCs) of host animal are the ones that interact most with probiotics^29^. Pattern recognition receptors are key components in the interaction and response of IECs and DCs to intestinal microorganisms^30,31^. In addition, probiotics could also exert immunomodulatory effects through interactions with monocytes/macrophages and lymphocytes^29^. A study of the effects of compound probiotics (*Lactobacillus*, yeast, *Bacillus*, etc.) on the immunity and antioxidant function of cows showed that the serum IgG content increased significantly in the high-dose probiotic-treated group^32^. In this study, as shown in Fig. 2, the increase in IgA and IgG levels in treatment group B was significantly higher than that in the control group (*p* < 0.05). The results showed that FMDPs might be beneficial for improving the concentrations of immunoglobulin in FMD. This result was in consistent with other similar studies, which showed that compound probiotics could increase the levels of immune factors in the serum of weaned calves and enhance the cellular immune function of the immune system^33^. Similarly, SUN *et al*.^34^ reported that the *B. subtilis* natto could increase the serum immunoglobulin levels of weaned calves and indicated that the probiotic *B. subtilis* could enhance the immune function of the immune system in calves.

CUI *et al*.^35^ explored the effects of a complex probiotic preparation on performance, fecal bacterium population and immunoglobulin in weaned fawns and showed that the increase in the fecal *Lactobacillus* population in the test group was more notable than that in the control group at the end of the test period (*p* < 0.05). Another study indicated that the lactic acid bacteria number in the feces of cows was significantly higher than that in the control group^36^. In this study, after FMDP feeding, the number of lactic acid bacteria in the feces of FMD increased significantly (Fig. 3).

To further explore the effect of microecological agents on the intestinal flora, we characterized the fecal microbiota of FMD using high-throughput 16S rRNA sequencing technology. As shown in Fig. 4, after FMDP feeding, the number of components shared by the control group and treatment group A increased, and the number of components shared by the control group and treatment group B also increased. The number of unique OTUs in the control group decreased, and the number of unique OTUs in the treatment groups increased. This situation may be due to the similarity in living environments and management methods. The number of shared OTUs increased, but the number of unique OTUs in the control group decreased, and the number of unique OTUs in the treatment groups fed FMDPs increased. This result indicated that the biodiversity in the intestinal tract of FMD increased after FMDP feeding.

Biodiversity and richness play an crucial role in maintaining the host’s normal physiological functions^37^. The alpha diversity was significantly different between the control group and treatment group B, and there were no significant differences between the control group and treatment group A. As shown in Fig. 5, the Chao1 index of treatment group B increased after FMDP feeding (*p* < 0.05), and the Shannon and Simpson indices of treatment group B also both increased after FMDP feeding (*p* < 0.01). Therefore, the results indicated that the biodiversity and richness in the gut of FMD increased after FMDP feeding (*p* < 0.05). Regarding beta diversity, on day 0 and day 30, the samples from the control group and the treatment groups were scattered. On day 60, most of the samples from the treatment groups were clustered together. The differences between treatment groups A and B decreased after FMDP feeding (Fig. 6). There was no significant difference observed between the two treated groups in terms of ADG, perhaps because the community structures of the two treatment groups became similar after FMDP feeding.

Interestingly, although the dominant fecal microbes in the control group and treatment groups were *Firmicutes* and *Bacteroidetes*, in this study, the F/B ratio increased in treatment group B (*p* < 0.05), and the F/B ratio was closely related to the weight. *Firmicutes* and *Bacteroidetes* are the predominant fecal microbes in human beings, participating mainly in the regulation of fat and bile acid metabolism and maintenance of the energy balance of the host^38^. Notably, *Firmicutes* and *Bacteroidetes* were the main drivers of polysaccharide fermentation in other studies^39–41^. In obese individuals, the F/B ratio is on the high side; the abundance of *Bacteroidetes* in fat individuals is low, but the abundance of *Firmicutes* is high^41^. In particular, when the F/B ratio in the gut increases, the host’s ability to absorb energy from food increases, as does the storage of fat in the body. Bäckhed *et al*.^42^ found that when mice were inoculated with polymorphous *Bacteroidetes* after sterilization, their body fat content increased significantly by 23%, but the increase was less than that observed upon inoculation with mixed cecal microorganisms (containing a high proportion of *Bacteroidetes*), which indicated that *Bacteroidetes* could promote fat deposition. This result might be related to the ability of *Bacteroidetes* to decompose plant polysaccharides, but promotion of fat deposition was not as strong as that exhibited by microbial communities with a high abundance of *Firmicutes*. *Firmicutes* and *Bacteroidetes* might have a synergistic symbiotic relationship, and a high F/B ratio might facilitate the absorption/storage of energy by the host and help the host gain weight.

In this study, notably, the composition of the fecal microbiotas in growing FMD changed after FMDP feeding. On day 30, the relative abundance of *Proteobacteria* was lower than that of *Firmicutes* in the control group, while the relative abundance of *Proteobacteria* was higher than that of *Firmicutes* in the treatment groups. It is possible that intake of FMDPs could modulate the fecal microbiota but could not attain an equilibrium state, presumably because of insufficient time. This hypothesis is consistent with previous studies showing that the relative abundances of *Lactobacillus* species in broilers were reduced at 28 days of age and gradually reached a stable state from 28 to 42 days of age^43^. Li *et al*.^44^ found that the fecal microbiota of broiler chickens would constantly change with age on a daily basis. On day 60, *Actinomycetes* replaced *Verrucomicrobia* as the dominant microbes in treatment group B (Table 1, Fig. 7). *Actinomycetes* interact with the host at the energy, gene and material levels, forming a host-specific microecological system in the long process of coevolution^45^. *Actinomycetes* participate in a series of important physiological activities such as host metabolism and maintenance of the intestinal microecological balance by producing various active substances (antibiotics, immunosuppressants, vitamins and enzymes) and promote the growth of animals^46,47^. At the genus level, after FMDP feeding, the relative abundances of *Ruminococcus*, *Akkermansia*, *Oscillospira*, *Coprococcus* and *Clostridium* were significantly increased in treatment group B on day 60 (*p* < 0.05) (Table 2). In a liver cirrhosis model, Xie *et al*.^48^ found that the levels of *Akkermansia*, *Coprococcus* and *Oscillospira* were significantly negatively correlated with pathophysiological indices, including blood glucose levels, blood lipid levels, plasma total bile acid levels and total bile acid levels. As an important member of gut probiotics, *Akkermansia* plays a key role in maintaining digestive tract barrier function, metabolic inflammation and fat storage in humans^49^. *Coprococcus* is closely related to pectin degradation in roughage, which could improve animal production performance by promoting the synthesis of rumen microbial proteins^50^. The relative abundance of *Coprococcus* was significantly increased in the treatment groups; therefore, the F/G ratios in both treatment groups A and B were significantly lower than that in the control group, finally causing a significant increase in ADG. *Clostridium* is the main bacterial genus that produces butyric acid, which is produced by microbial fermentation and plays an important role in maintaining host health and preventing disease^51^. In the host colon epithelium, *Clostridium* could provide an optimal source of carbon and nitrogen to promote the growth of IECs and could accelerate the repair of the damaged intestinal mucosa. Previous studies demonstrated that *Clostridium* promoted the growth of *Lactobacillus* and *Bifidobacterium* and inhibited antibiotic-associated diarrhea^52–57^ in humans and mice. Additionally, Zhang *et al*.^58^ reported that *Clostridium* might have beneficial effects on the immune system.

The relative abundances of *Lactobacillus* species were increased but not significant. It is possible that the metabolites of *Lactobacillus* can inhibit the activity of pathogenic bacteria but have no influence on the surrounding probiotics; thus, the number of beneficial bacteria (*Coprococcus*, *Akkermansia*, etc.) increased substantially. The number of *Lactobacillus* species and beneficial bacteria increased at the same time, causing no significant difference in the relative abundance of *Lactobacillus*. The number of probiotics increased, the F/G ratio was reduced, and the ADG increased eventually.

Under the conditions of this experiment, the addition of FMDPs in the diets could increase the ADG of FMD by reducing the F/G ratio and increasing the feed conversion ratio. The addition of FMDPs changed the community structure of the FMD microbiota and increased the number of beneficial bacteria in the intestinal tract of FMD. FMDPs tended to increase the immunoglobulin content in FMD, which was beneficial for enhancing the immune function of the body. Hence, FMDPs have broad application prospects in the regulation of the normal microbial flora in the digestive tract of FMD, and this study laid a foundation for exploring the mechanism underlying the effect of FMDPs on the weight, immunity and intestinal flora of FMD.

## Materials and Methods

### Animals and feeding management

Twelve healthy seven-month-old growing FMD from the Ma’erkang musk deer breeding farm in Sichuan, China, were randomized into three groups, named control group, treatment group A and treatment group B. The control group was fed a basal diet. Treatment group A was fed the basal diet supplemented with 10^10^ colony-forming units (CFU)/mL FMDPs, which was stored at the Animal Quarantine Laboratory, Sichuan Agricultural University, in Sichuan, China, and composed of *Lactobacillus plantarum*, *Lactobacillus acidophilus* and *Leuconostoc* species. Treatment group B was fed the basal diet supplemented with 2** × **10^10^ CFU/mL FMDPs. The pretrial period lasted for 7 days, and the experiment lasted for 60 days. During the experiment, all groups had free access to food and water, and daily feed intake was recorded. This experiment was carried out under appropriate illumination and ventilation conditions and with proper feeding and management.

### Sample collection

Fresh fecal samples were collected in the morning on day 0, day 30, and day 60. Samples were collected with sterile gloves and stored immediately after collection in a sterile centrifuge tube that was sealed to avoid cross-contamination among samples. After sampling, half of the samples were frozen in liquid nitrogen and stored at −80 °C in the laboratory for DNA extraction. The other half were stored with an ice pack and returned to the laboratory. Blood samples of all FMD were collected from each group 2 h before the morning feeding. The serum was prepared by taking approximately 5 mL of blood and centrifuging for 10 min at 3,000 rpm in a centrifuge. All serum samples were marked according to groups and stored at −20 °C^59^.

### Determination of body weight

Growing FMD were weighed individually on day 0, day 30, and day 60 to determine ADG 2 h before the morning feeding. The weights were recorded and used to make the growth curve.

### Determination of the number of lactic acid bacteria in feces

The viable counts of lactic acid bacteria in feces were determined by plating 20 μL of the 10-fold serially diluted fecal samples (0.9% NaCl) on MRS agar in triplicate followed by incubation at 37 °C for 24 h. The average number of CFU from the three plates was used to calculate the concentration of lactic acid bacteria in the culture.

### Determination of immune-related factors

The serum was prepared for determination of serum immune indicators (IgA, IgG, IgM, IFN-γ, TNF-α and IL-2) by using an ELISA Kit (Nanjing SenBeiJia Biological Technology Co., Ltd.), and specific steps, measurement and calculation were performed based on the operation manual^60^.

### Statistical analysis

The test data were examined using the SPSS 20.0 software package for single-factor analysis of variance, and the results were shown as the mean ± standard deviation with *p* < 0.05 as the criterion for significant difference.

### DNA extraction and purification

Based on the operation manual, the acquisition of total genomic DNA was performed using the QIAamp DNA Stool Mini Kit (QIAGEN, Hilden, Germany) from fecal samples and the DNA was ready for the sequencing. The molecular size determination was performed by agarose gel electrophoresis (0.8% gel), and the DNA was estimated with a NanoDrop 2000 spectrophotometer (Thermo Fisher Scientific, Waltham, MA, USA).

### 16S rRNA amplicon pyrosequencing

In this experiment, the highly variable V3-V4 region of the bacterial 16S rRNA gene was amplified with the forward primer 338F (5′-ACTCCTACGGGGGCAGCA-3′) and reverse primer 806R (5′-GGACTACHVGGGTWTCTAAT-3′). Sample-specific 7-bp barcodes were added to the primer. The PCR mixtures contained 12.25 μL of the PCR Mix, 1 μL (10 μmol/L) of the forward and reverse primers, 2 μL of DNA template, and 8.75 μL of ddH_2_O. Amplification reaction conditions: pre-denaturation at 98 °C for 2 min; denaturation at 98 °C for 15 s, annealing at 55 °C for 30 s, extension at 72 °C for 30 s, followed by 25 cycles and extension at 72 °C for 5 min. The PCR amplicons were purified using Agencourt AMPure beads (Beckman Coulter, Indianapolis, IN) and quantified using the PicoGreen dsDNA Assay Kit (Invitrogen, Carlsbad, USA)^61–63^.

### Bioinformatics and statistical analysis

Paired-end sequencing of the microbial DNA fragments was detected by the Illumina MiSeq platform. The obtained sequences with 97% similarity were classified into OTUs and merged sequences, and representative sequence was the sequence with the highest abundance in each OUT. QIIME and R packages (v3.2.0) were used for the sequence data analyses. The alpha diversity indices at the OTU level were calculated in QIIME. Chao1 and ACE are the major indices of species richness in ecology. The Chao1 and ACE indices are in direct proportion to the richness of the community. The Shannon index comprehensively considers the richness and uniformity of the community, and the Simpson index is one of the most commonly used indices for evaluating community diversity. The Shannon and Simpson indices are in direct proportion to the diversity of the community. Beta diversity analysis was used mainly to determine the difference of species composition among all samples by principal component analysis (PCA), multidimensional scaling (MDS) and clustering analysis methods. PCA was performed to quantify the differences and similarities among samples by linear transformation^64^. A Venn diagram was used to show the core fecal microbiota of each sample or group by the “Venn Diagram”^65^. Based on the composition and sequence distribution of the samples at each taxonomic level, the abundance differences among samples or groups could be compared separately, and the significance of the difference was tested by Metastats^66^. Linear discriminant analysis effect size (LEfSe) was used to examined the taxa with significant differences among groups by the default parameters^67^.

### Ethics approval

All experiments animals are managed according to the guidelines for the care and use of lab animals and approved by the Committee on Experimental Animal Management of the Sichuan Agricultural University (Approval No. SYXK2019-187).

## References

[1] Sahadeva R, Leong S, Chua K (2011). Survival of commercial probiotic strains to pH and bile. International Food Research Journal..

[2] Gerritsen J, Smidt H, Rijks GT, Vos WM (2011). Intestinal microbiota inhuman health and disease: the impact of probiotics. Genes Nutrition..

[3] Fooks LJ, Gibson GR (2002). Probiotics as modulators of the gut flora. Br J Nutr..

[4] Ng SC (2009). Mechanisms of action of probiotics: recent advances. In flam Bowel Dis..

[5] Fukuda S (2011). Bifidobacteria can protect from enteropathogenic infection through production of acetate. Nature..

[6] Mao-wen L (2014). Effects of Chromium Picolinate on Body Temperature, Respiration Rate, Milk Yield and Composition of Cattle under Heat Stress. Feed & Feeding..

[7] Marwati, T. *et al*. Inhibitory activity of bacteriocin produced from *Lactobacillus* SCG 1223 toward L. *monocytogenes*, S. *thypimurium* and E. coli. IOP Conf. Series: Earth and Environmental Science. **102** (2018).

[8] Balsari A (1982). The fecal microbial population in the irritable bowel syndrome. Microbiology..

[9] Nadja L (2018). Characterization of *Bacillus spp*. strains for use as probiotic additives in pig feed. Applied microbiology and biotechnology..

[10] Marsalková S (2004). Testing two *Lactobacillus plantarum* and *Lactobacillus acidophilus* strains for their suitability as a lipoid probiotic. Berl Munch Tierarztl Wochenschr..

[11] Keles G, Demirci U (2011). The effect of homofermentative and heterofermentative lactic acid bacteria on conservation characteristics of baled triticale-Hungarian vetch silage and lamb performance. Animal Feed Science and Technology..

[12] Zhang, E. Musk deer. In: Wemmer, C. (ED). Deer, Status Survey and Conservation Action Plan. IUCN/SSC Deer specialist Group, IUCN, Gland, Switzerland and Cambridge. *UK, pp*. 72–76 (1998).

[13] Yang Q, Meng X, Xia L, Feng Z (2003). Conservation status and causes of decline of musk deer (Moschus spp.) in China. Conserv..

[14] Wang, Y. & Harris, R. Moschus berezovskii (errata version published in 2016). The IUCN Red List of Threatened Species 2015: e.T13894A103431781 (2015).

[15] Qi WH (2011). The reproductive performance of female Forest musk deer (Moschus berezovskii) in captivity. Theriogenology..

[16] Meng Xiuxiang *et al*. Musk deer farming in China. Cambridge University Press. pp. 1–6 (2006).

[17] Wang HY, Cai YH, Chen JG (2009). Study on the reproductive parameters of captive Forest musk deer. China Herbivores..

[18] Luo X (2014). Development of Compound Probiotic for Forest Musk Deer. Sichuan Journal of Zoology..

[19] DeSantis TZ (2006). Greengenes, a chimera-checked 16S rRNA gene database and workbench compatible with ARB. Appl Environ Microbiol..

[20] Ramette A (2007). Multivariate analyses in microbial ecology. FEMS Microbiol Ecol..

[21] Standen, Benedict. The effect of dietary probiotics on Nile tilapia, Oreochromis niloticus, health and growth performance. UK: *University of Plymouth* (2015).

[22] Liu, L. *et al*. Probiotic *Clostridium butyricum* Improves the Growth Performance, Immune Function, and Gut Microbiota of Weaning Rex Rabbits. *Probiotics Antimicrob Proteins*. 1–15 (2018). 10.1007/s12602-018-9476-x 30324399

[23] Yunqin FU (2012). Effects of Different Combinations of Probiotics on Growth Performance and Serum Biochemical Parameters in Dairy Calves Aged from 0 to 8 Weeks. Chinese Journal of Animal Nutrition..

[24] Jia, P. *et al*. Influence of dietary supplementation with *Bacillus licheniformis* and Saccharomyces cerevisiae as alternatives to monensin on growth performance, antioxidant, immunity, ruminal fermentation and microbial diversity of fattening lambs. *Sci Rep* (2018). 10.1038/s41598-018-35081-4 PMC6232095 30420720

[25] Steven M (1988). Probiotics: Intestinal inoculants for production animals. Vet. Med..

[26] Schiffrin EJ (1995). Immunomodulation of human blood cells following the ingestion of lactic acid bacteria. J. Dairy Sci..

[27] Kabir SML (2004). The dynamics of probiotics on growth performance and immune response in broilers. Int. J. Poult. Sci..

[28] Mountzouris KC (2010). Effects of probiotic inclusion levels in broiler nutrition on growth performance, nutrient digestibility, plasma immunoglobulins, and cecal microflora composition. Poult. Sci..

[29] Bermudez BM (2012). Probiotic mechanisms of action. Ann NutrMetab..

[30] Gómez LC, Muñoz S, Gil A (2010). Role of Toll-like receptors in the development of immunotolerance mediated by probiotics. Proc Nutr Soc..

[31] Lebeer S, Vanderleyden J, De Keersmaecker CJ (2010). Host interactions of probiotic bacterial surface molecules: comparison with commensals and pathogens. Nat Rev Microbiol..

[32] Xiaozheng FU (2014). Effects of complex probiotics on immune and antioxidative function in dairy cattle. Cereal & Feed Industry..

[33] Qadisa Q (2014). Effect sofa bacteria-based probiotic on ruminal pH, volatile fatty acids and bacterial flora of Holstein calves. Vet Med Sci.

[34] Sun P, Wang JQ, Zhang HT (2010). Effects of Bacillus Subtilis natto on performance and immune function of preweaning calves. Dairy Sci..

[35] Yi-zhe, C. *et al*. Effects of complex probiotic preparation on performance, fecal bacterium population and immunoglobulin in weaned fawns. *Chinese Journal of Veterinary Science* (2017).

[36] Xiaozheng FU (2015). Effect of Complex Probiotics on Ammonia Production and Microorganisms in Cattle Manure. Acta Ecologae Animalis Domastici..

[37] Koboziev I (2014). Role of the enteric microbiota in intestinal homeostasis and inflammation. Free Radic. Biol. Med..

[38] Tum PJ (2006). An obesity-associated gut Microbiome with increased capacity for energy harvest. Nature..

[39] Ley RE (2005). Obesity alters gut microbial ecology. Proceedings of the National Academy of Sciences of the United States of America..

[40] Toru M (2012). Antibiotics production by an actinomycete isolated from the termite gut. Journal of Basic Microbiology..

[41] Ley RE (2006). Microbial ecology: human gut microbes associated with obesity. Nature..

[42] Bäckhed F (2004). The gut microbiota as an environmental factor that regulates fat storage. Proceedings of the National Academy of Sciences of the United States of America..

[43] Xie G (2016). Distinctly altered gut microbiota in the progression of liver disease. Oncotarget..

[44] Li K (2015). Effects of Different Probiotics on Intestinal Microbial Community Structure of Broiler. Chinese Journal of Animal Nutrition.

[45] Jin HZ, Li KB (2004). Progress in the study of human intestinal microecosystem. Nature Magazine.

[46] Gandhimathi R (2008). Antimicrobial potential of sponge associated marine actinomycetes[J]. Journal of Medical Mycology.

[47] Jiang Y (2012). Diversity and bioactivity of culturable actinobacteria from animal feces. Acta Microbiologica Sinica..

[48] Xie, W. H. Effects of Compound Probiotics on Growth, Immunity Indices and Intestinal Flora in Broilers. Heilongjiang: *Heilongjiang Bayi Agricultural University* (2018).

[49] Everard, A. *et al*. Cross-talk between *Akkermansia muciniphila* and intestinal epithelium controls diet-induced obesity. *PNAS* (2013). 10.1073/pnas.1219451110 PMC3670398 23671105

[50] Liu, W. Isolation and identification of a lactate-utilizing, butyrate-producing bacterium from procine feces and its metabolic characteristics in virtrol. Nanjing: *Nanjing Agricultural University* (2007). 17672301

[51] Jang YS (2013). Metabolic engineering of *Clostridium acetobutylicum* for enhanced production of butyric acid. Appl Microbiol Biotechnol..

[52] Kamiya S (1997). Bacterioprophylaxis using *Clostridium butyricum* for lethal caecitis by *Clostridium difficile*. Rev. Med. Microbiol..

[53] Imase K (2008). Efficacy of *Clostridium butyricum* preparation concomitantly with Helicobacter pylori eradication therapy in relation to changes in the intestinal microbiota. Microbiol Immunol..

[54] Seki H (2003). Prevention of antibiotic-associated diarrhea in children by *Clostridium butyricum* MIYAIRI. Pediatr. Int..

[55] Kong Q (2011). Oral administration of *Clostridium butyricum* for modulating gastrointestinal microflora in mice. Current. Microbiol..

[56] Zhang B, Yang X, Guo YM, Long FY (2011). Effects of dietary lipids and *Clostridium butyricum* on the performance and the digestive tract of broiler chickens. Arch. Anim. Nutr..

[57] Yang. CM (2012). Effects of probiotic, *Clostridium butyricum*, on growth performance, immune function, and cecal microflora in broiler chickens. Poultry Science..

[58] Zhang. L (2014). Effects of *Clostridium butyricum* on growth performance, immune function, and cecal microflora in broiler chickens challenged with Escherichia coli K88. Poultry Science.

[59] Zhang MS (2018). Comparative Analysis of Gut Microbiota Changes in Père David’s Deer Populations in Beijing Milu Park and Shishou, Hubei Province in China. Front Microbiol..

[60] Nonnecke BJ (2012). Adaptive immunity in the colostrum-deprived calf: Response to early vaccination with *Mycobacterium bovis* strain bacille Calmette Guerin and ovalbumin. J. Dairy Sci..

[61] Laursen MF (2017). Genomic GC-Content Affects the Accuracy of 16S rRNA Gene Sequencing Based Microbial Profiling due to PCR Bias. Frontiers in Microbiology..

[62] Emily MG (2018). Evaluating Established Methods for Rumen 16S rRNA Amplicon Sequencing With Mock Microbial Populations. Frontiers in Microbiology..

[63] Fengjie Huang (2019). Theabrownin from Pu-erh tea attenuates hypercholesterolemia via modulation of gut microbiota and bile acid metabolism. Nature. Communications..

[64] Heck KL, van Belle G, Simberloff D (1975). Explicit Calculation of the Rarefaction Diversity Measurement and the Determination of Sufficient Sample Size. Ecology..

[65] Zaura E (2009). Defining the healthy “core microbiome” of oral microbial communities. Bmc Microbiology..

[66] White JR, Nagarajan N, Pop M (2009). Statistical Methods for Detecting Differentially Abundant Features in Clinical Metagenomic Samples. Plos Computational Biology..

[67] Segata N (2011). Metagenomic biomarker discovery and explanation. Genome Biology..

